# Effects of a coal to clean heating policy on acute myocardial infarction in Beijing: a difference-in-differences analysis

**DOI:** 10.1016/S2542-5196(24)00243-2

**Published:** 2024-11-05

**Authors:** Martha Lee, Jie Chang, Qiuju Deng, Piaopiao Hu, Honor Bixby, Sam Harper, Guofeng Shen, Shu Tao, Moning Guo, Feng Lu, Jill Baumgartner, Jing Liu

**Affiliations:** a Department of Epidemiology, Biostatistics, and Occupational Health, School of Population and Global Health, McGill University, Montrael, QC, Canada; b Department of Equity, Ethics and Policy, School of Population and Global Health, McGill University, Montrael, QC, Canada; c Center for Clinical and Epidemiologic Research, Beijing An Zhen Hospital, Capital Medical University, Beijing, China; d Beijing Institute of Heart, Lung and Blood Vessel Diseases, Beijing, China; e Institute of Public Health and Wellbeing, University of Essex, Colchester, UK; f Laboratory for Earth Surface Processes, College of Urban and Environmental Sciences, Peking University, Beijing, China; g Beijing Municipal Health Big Data and Policy Research Center, Beijing Institute of Hospital Management, Beijing, China

## Abstract

**Background:**

In 2015, the Chinese Government launched the coal to clean heating policy (CHP), designed to improve air quality and health in China. The CHP banned household coal burning and provided subsidies for clean electric or gas-powered heating for millions of peri-urban and rural households. We aimed to investigate whether the CHP affected the incidence of acute myocardial infarction in Beijing townships.

**Methods:**

In this quasi-experimental study, we obtained township data on acute myocardial infarction hospital admissions and deaths, exposure to the CHP (yes *vs* no), and a range of covariates for periods before (Jan 1, 2013, to Dec 31, 2014) and after the CHP began (Jan 1, 2016, to Dec 31, 2017; and Jan 1, 2018, to Dec 31, 2019). The policy was gradually rolled out across villages, and townships in our study were considered exposed to the policy in periods when more than 50% of their villages were assigned into the CHP. We estimated the effect of the CHP on township incidence of acute myocardial infarction for all adults (aged ≥35 years) and separately for sex and older adults (aged ≥65 years) using a difference-in-differences approach that accommodates the progressive roll-out of the policy.

**Findings:**

Of 307 townships in Beijing, we excluded 156 (51%) urban townships where most villages had central heating and were thus ineligible for the CHP. Of the 151 peri-urban and rural Beijing townships considered eligible for the CHP, 75 (50%) townships were exposed to the CHP by the end of 2017 and 92 (61%) by the end of 2019. We estimated an overall reduction of 6·6% (95% CI –12·3 to –0·8) in the incidence of acute myocardial infarction from before to after roll-out of the CHP in exposed townships relative to those not exposed to the policy, with some evidence of larger effects in women (–11·7% [–19·0 to –4·1%]), older adults (–10·7% [–17·4 to –3·6%]), and in townships exposed for longer (–3·5% [–9·5 to 2·8%] after <2 years and –9·7% [–18·3 to –0·5%] after 2–4 years).

**Interpretation:**

Our results provide among the first empirical evidence of possible cardiovascular benefits from a household clean energy policy, and support efforts to implement and assess such policies in China and globally.

**Funding:**

Wellcome Trust, the Canadian Institutes for Health Research, and the National Natural Science Foundation of China.

## Introduction

Cardiovascular disease is the leading cause of death in China, accounting for approximately two of every five deaths in 2019 and imposing large economic and social burdens.^1^ Acute myocardial infarction is a common and serious form of cardiovascular disease that is often fatal. Mortality from acute myocardial infarction in China has increased by five times over the past two decades,^1^ making prevention of acute myocardial infarction a public health priority.

An estimated 1·1 million (24%) of China's 4·6 million cardiovascular deaths in 2019 were attributed to air pollution and use of solid fuels (eg, biomass and coal) for cooking and heating.^2^ Short-term exposure to air pollution is associated with cardiovascular deaths and non-fatal events including acute myocardial infarction, and long-term exposure further increases risk of cardiovascular mortality.^3^ The use of solid fuel stoves is also associated with increased blood pressure and higher risk of cardiovascular mortality,^4^ with several randomised trials showing that switching to clean fuel for cooking has a protective effect on blood pressure in older adults.^5^ Collectively, these studies indicate that policies to reduce household solid fuel use might offer a powerful opportunity to reduce a population's cardiovascular disease burden.^6^

In 2015, the Chinese Government launched an ambitious rural clean heating policy (CHP) to reduce air pollution emitted from coal-burning stoves and boilers in northern China (appendix p 2). Coal combustion comprised over 70% of household heating energy in northern China before the CHP,^7^ contributing to high exposures to household air pollution^8^ and around 30% of wintertime outdoor air pollution.^9^ Between 2017 and 2021, the CHP progressively transitioned over 36 million coal-burning households to more efficient electric or gas-powered heating by banning residential coal burning and subsidising the costs of replacement heaters and electricity.^10^, ^11^ Whether this large-scale energy policy yielded health benefits remains an important and unresolved question. We therefore studied the effects of the CHP on township incidence of acute myocardial infarction in Beijing from 2013 to 2019 using a difference-in-differences (DiD) approach with multiple time periods.

**Research in context**

**Evidence before this study**

Previous studies have established air pollution and household use of solid-fuel stoves as risk factors for cardiovascular mortality and non-fatal cardiovascular events, although evidence on the effects of solid-fuel use is scarcer. Many countries have implemented household energy policies and programmes to replace solid-fuel stoves with less polluting alternatives. Such policies are potentially important for cardiovascular health; however, there is little empirical evidence for the cardiovascular benefits of clean energy transition beyond some randomised and non-randomised cookstove intervention studies showing protective effects on blood pressure. We searched PubMed for articles published from inception to Dec 12, 2023, reporting on the cardiovascular effects of household energy policies or programmes, using the search terms (“household fuel use” OR “coal” OR “fuel use”) AND (“quasi-experiment” OR “evaluation study” OR “program evaluation” OR “policy evaluation”). We also searched the peer-reviewed literature using the names of policies listed in the WHO Household Energy Policy Repository. We retrieved four relevant studies. Residential wood-burning or coal-burning regulations or bans were associated with fewer cardiovascular hospitalisations in California, USA (although this study did not control for secular changes in health), and with reduced cardiovascular mortality in Tasmania, Australia; but no changes in cardiovascular mortality were observed in Ireland following a residential coal ban. In northern China, a cohort analysis of data from 8524 adults across 11 cities where the clean heating policy (CHP) was piloted and 21 cities not included in the pilot found no difference in physician-diagnosed cardiovascular disease in pilot versus non-pilot cities. However, this study was limited by a short follow-up period (1 year) and by possible confounding by other city-level health-related policies or programmes.

**Added value of this study**

We investigated whether implementation of northern China's CHP, one of the most ambitious clean household energy policies to date, affected the incidence of acute myocardial infarction in Beijing townships. Unlike the few previous assessments of household energy policies, our quasi-experimental design better controls for potential biases from secular trends and for potential confounding by time-varying risk factors for acute myocardial infarction that could evolve differently in townships implementing or not implementing the CHP. Our findings indicate a potential benefit of the CHP on the incidence of acute myocardial infarction in townships adopting the CHP, with some evidence of larger effects among women, older adults, and townships adopting the CHP for longer. This study makes an important contribution to the evidence on household energy interventions, which is comprised of observational studies assessing cardiovascular outcomes for users of different fuel and stove types and a few small-scale cookstove intervention studies that assessed subclinical cardiovascular endpoints such as blood pressure.

**Implications of all the available evidence**

Household solid-fuel use was responsible for an estimated 2·3 million global deaths in 2019, including over a million cardiovascular deaths. Provision of clean household energy is a potential intervention to reduce cardiovascular burden in settings where household solid-fuel use is common. Our study's findings support the conclusions of the small number of randomised and non-randomised intervention studies showing a protective effect of clean cookstoves on blood pressure in older women, and provides a methodologically robust contribution to the studies (mostly in high-income countries) showing cardiovascular benefits of household energy policies.

## Methods

### Study design and participants

Beijing, the capital of China, covers a large geographical area (16 410 km^2^) with a densely populated and developed urban core that is surrounded by satellite towns and rural agricultural areas. The city's 16 administrative districts are further divided into townships that contain rural and urban villages. Winters are dry and cold (January average –3·9°C),^12^ thus requiring indoor room heating. Most urban Beijing households are connected to central heating and use portable electric-powered heaters, whereas peri-urban and rural households historically relied on coal-fuelled and raw biomass-fuelled heaters and cookstoves.^7^

We used a quasi-experimental design to measure the effect of the CHP on the incidence of acute myocardial infarction in Beijing townships, the highest spatial resolution for which acute myocardial infarction data were available. We excluded townships in the urban core or satellite towns where most villages would be ineligible for the CHP because they accessed central heating (appendix p 2). The protocol for acquisition and analysis with acute myocardial infarction data was approved by the ethics review committee at Beijing An Zhen Hospital, Capital Medical University (2021139X).

### CHP participation

**Figure 1:**
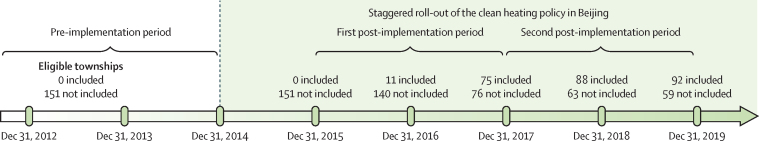
Study timeline

We obtained a list of villages (n=2509) that were enrolled into the CHP between 2015 and 2019 along with year of participation from the College of Urban and Environmental Sciences at Peking University and matched values to a geolocated database of all Beijing villages obtained from the China National Bureau of Statistics (appendix p 2). We calculated the proportion of enrolled villages in each township and time period, and created a binary inclusion variable that considered eligible townships as exposed to the CHP if most of their villages (>50%) were enrolled into the policy in a given time period, and as not exposed to the CHP otherwise (figure 1).

### Incidence of acute myocardial infarction

The outcome of interest for this analysis was township-level incidence of acute myocardial infarction (ie, the number of acute myocardial infarction events per 100 000 population in each study period) among permanent residents aged 35 years and older. Acute myocardial infarction events for the years 2007–19 were extracted from the Beijing Cardiovascular Disease Surveillance System, which records all out-of-hospital deaths for acute myocardial infarction and secondary-level and tertiary-level hospital admissions, along with patient age, sex, date of onset, and residential address (appendix p 2). Diagnosis was based on principal discharge diagnoses or underlying cause of death with ICD-10 codes I21–22 (acute myocardial infarction and subsequent myocardial infarction). Population data were obtained from district statistical yearbooks. We calculated age-standardised and sex-standardised rates using the 2010 Beijing population as the reference (appendix p 3).

We fitted Bayesian spatial models to estimate township-level incidence of acute myocardial infarction for all adults and separately by sex and for older adults (aged ≥65 years) for time periods before (Jan 1, 2013, to Dec 31, 2014) and after the introduction of the CHP (Jan 1, 2016, to Dec 31, 2017, and Jan 1, 2018, to Dec 31, 2019) for 307 townships, using methods described in the appendix (p 3) and published elsewhere.^13^ Additionally, we estimated the acute myocardial infarction rates for Jan 1, 2007, to Dec 31, 2008; Jan 1, 2009, to Dec 31, 2010; and Jan 1, 2011, to Dec 31, 2012, to assess temporal trends by eventual CHP exposure cohort before the policy was implemented. We pooled acute myocardial infarction events over 2 years to create more stable estimates for less-populated townships and sex–age subgroups. We excluded 2015, the pilot year of the CHP, because only 18 villages were exposed to the policy and no townships met our definition of CHP exposure (ie, more than 50% of villages in the CHP). We did not separately estimate township incidence of acute myocardial infarction for adults aged 35–64 years because events were less common in this age range and evidence of pre-policy parallel trends was weak.

### Covariates

We created a database of covariates that are established risk factors for acute myocardial infarction.^6^, ^14^, ^15^ Pre-implementation socioeconomic variables from the 2010 national census included the proportion of the population in each township working in agriculture, having completed secondary school education or higher, and unemployed. We also obtained time-varying data on township prevalence of tobacco smoking, obesity, and hypercholesterolaemia; variability in annual and wintertime outdoor temperature; health-care access; and exposure to a retired coal-burning power plant during the study period (appendix p 4). Most of the Beijing population is of Han Chinese ethnicity (96%) according to the 2010 census, so ethnicity was not included as a covariate.

### Statistical analysis

We calculated the means (SD) and medians (IQR) of acute myocardial infarction incidence and covariates for groups over time. We used a DiD analysis for staggered CHP roll-out to compare the incidence of acute myocardial infarction in townships considered exposed to the CHP with the incidence of acute myocardial infarction in townships considered as not exposed (appendix p 6).^16^ Briefly, these methods allow for the estimation of causal effects (under specific assumptions) of CHP changes enacted at the group level by comparing the change in acute myocardial infarction incidence over time in CHP-exposed townships with the change in townships not exposed to the policy, under an assumption that, in the absence of the CHP, the counterfactual path of acute myocardial infarction incidence in exposed townships is the same as the observed path of acute myocardial infarction incidence in unexposed townships. We used the approach introduced by Callaway and Sant'Anna^16^ for DiD with multiple time periods. This approach allows for the effect of the CHP to vary over time (ie, the effect of the CHP on health might change with duration of exposure), which allowed us to estimate the dynamic average treatment effect on the treated (ie, the ATT), which is a contrast of the post-intervention outcomes in townships exposed to the policy with the counterfactual estimate of outcomes in the same population in the absence of the policy, where we estimate the effect of the policy by length of CHP exposure (ie, group–time effects after <2 years and 2–4 years of exposure) and an overall ATT that aggregates the dynamic group–time effects.^16^ Although we primarily focused on the overall (aggregate) ATTs, we also used omnibus joint F-tests and tests of interaction to assess whether evidence sufficed to reject the assumption of equality across CHP exposure cohorts and sex–age groups.

We conducted the DiD analyses with and without covariates for all adults and separate sex–age groups using a doubly robust estimation that combines outcome regression with inverse probability weighting to calculate propensity scores that attempt to balance pre-CHP covariates between townships exposed and not exposed to the CHP and thus reduce the likelihood of confounding (appendix p 5).^16^ The covariate-adjusted DiDs included township prevalence of employment in agriculture, unemployment, completion of secondary school education or higher, tobacco smoking, and obesity; variability in annual daily outdoor temperature; health-care access; and exposure to a retired coal-fired power plant.

Township incidence of acute myocardial infarction was log-transformed to better approximate a normal distribution; thus, the ATT estimates are presented as the percentage change in incident acute myocardial infarction, calculated as (exp[coef] − 1)*100. To incorporate the uncertainty from small-area estimation of township acute myocardial infarction, we generated the 95% CIs for the ATTs by doing each DiD analysis 6000 times using the 6000 random draws of township incidence rates of acute myocardial infarction from the posterior distributions of the Bayesian model used to estimate acute myocardial infarction.^13^ We report the medians of the distributions of the upper and lower 95% CIs values calculated from all 6000 runs.

### Sensitivity analyses and robustness checks

We ran several alternate specifications of the models to assess the robustness of our results. First, we tested the effect of small changes to township acute myocardial infarction estimation including age-standardisation with smaller age groups (35–49, 50–64, 65–79, and ≥80 years), age-stratified analysis for older ages when acute myocardial infarction is most common (65–79 years and ≥80 years), pooling acute myocardial infarction events over 2-year periods starting in November, which better aligns with the Nov 15 start of the heating season, and by pooling events over 1-year periods for older adults (aged ≥65 years), to potentially better temporally align with the timing of CHP implementation. Second, we assessed the influence of outliers by excluding two townships with especially high incidence of high acute myocardial infarction that were visually identified using boxplots. Third, we excluded four townships not implementing the CHP that appeared to mostly comprise urban villages that were ineligible for roll-out of the CHP. Fourth, we used a more distinct specification of CHP exposure that defined “exposure to the policy” as more than 70% of villages enrolled into the CHP and “not exposed to the policy” as less than 30% of villages exposed to the CHP, which excluded 60 townships with 30–70% of villages in the CHP from the analysis. Finally, we additionally adjusted for township prevalence of hypercholesterolaemia, which could potentially be a confounder or along the casual pathway,^17^ and for variability in heating-season temperature rather than annual.

All analyses were done in R (version 4.2.2), using the R2OpenBUGS and did packages.

### Role of the funding source

The funders of the study had no role in study design, data collection, data analysis, data interpretation, or writing of the report.

## Results

Of 307 townships in Beijing in 2010, we excluded 156 (51%; appendix p 2) urban townships where most villages had central heating and were thus ineligible for the policy. Our final sample of 151 CHP-eligible peri-urban and rural townships represents 29% (5·7 million) of Beijing's 2010 population (20·0 million) and nearly all of Beijing's census-designated rural population (2·8 million).

**Figure 2:**
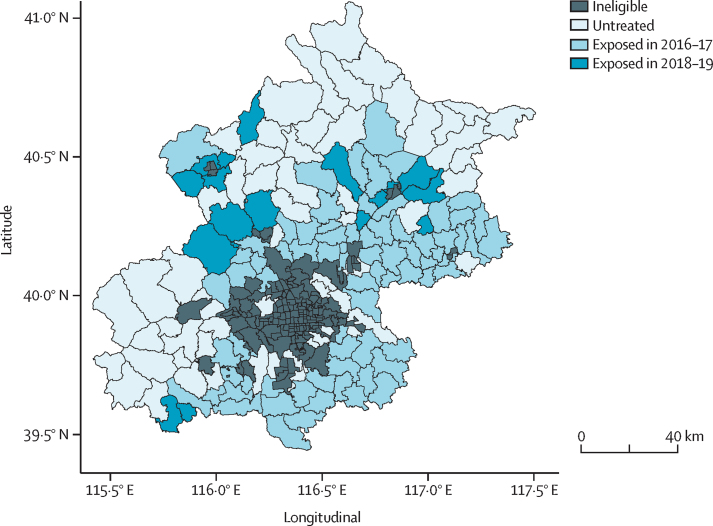
Township exposure to the clean heating policy in Beijing

Among the 151 eligible townships, the median proportion of villages exposed to the CHP by Dec 31, 2019, was 65% (IQR 30–89; appendix p 6). 75 townships were considered as exposed to the CHP by the end of 2017 and 92 by the end of 2019 (figure 1). Most townships included in the CHP were near the urban core and in the southeastern plains, with fewer in the western and northern areas of Beijing (figure 2).

Log-transformed mean and median township incidence of acute myocardial infarction for all adults and for different sex–age groups followed similar temporal trends by CHP exposure cohort in the 8 years before the CHP, supporting the key assumption of the DiD analysis that similar pre-CHP trends would have persisted over time in the absence of the CHP (appendix p 7). In the 2013–14 pre-CHP period, the median incidence of acute myocardial infarction was highest in townships exposed to the policy in 2016–17, followed by townships not exposed to the CHP, and finally in those exposed to the CHP in 2018–19 (table; appendix p 8). The distributions of covariates were similar across exposure cohorts, although townships not exposed to the CHP tended to have lower health-care access and educational attainment than exposed townships (table; appendix p 9). Time-varying covariates followed similar temporal trends, except for the township prevalence of obesity and smoking, where initial differences between exposure cohorts converged over time (appendix pp 9, 12–14).

**Table:** Township characteristics in the pre-exposure period (2013–14), by eventual clean heating policy exposure group

			**Unexposed (n=59)**	**Exposed in 2016–17 (n=75)**	**Exposed in 2018–19 (n=17)**
Incidence of acute myocardial infarction (events per 100 000 population)					
	Adults aged ≥35 years		266 (225–350)	325 (277–383)	248 (224–286)
		Men	327 (280–439)	406 (356–487)	309 (291–380)
		Women	192 (161–247)	226 (198–283)	177 (158–223)
	Adults aged ≥65 years		854 (694–1064)	1034 (878–1267)	798 (728–1015)
		Men	890 (773–1053)	1147 (927–1382)	907 (673–1026)
		Women	836 (641–1086)	939 (829–1262)	794 (652–1006)
Population working in agriculture			81% (70–88)	78% (73–83)	79% (65–82)
Population unemployed			4% (4–6)	4% (4–4)	4% (4–4)
Population with secondary school education or higher			24% (19–29)	27% (24–32)	28% (23–47)
Population currently smoking			25% (22–32)	24% (21–28)	31% (20–32)
Population with obesity*			23% (21–25)	21% (18–25)	23% (21–24)
Population with hypercholesterolaemia†			8% (5–8)	7% (6–8)	8% (4–8)
Health-care access (number of hospital beds per 1000 individuals)			1·4 (0·3–3·0)	2·4 (1·7–3·1)	1·9 (1·2–3·5)
Annual variability in daily outdoor temperature, °C			11·4 (11·3–11·6)	11·6 (11·5–11·7)	11·4 (11·3–11·6)
Heating season‡ variability in daily outdoor temperature, °C			3·3 (3·1–3·4)	3·0 (3·0–3·2)	3·2 (3·1–3·4)

Data are median (IQR). Townships were considered exposed to the clean heating policy if over 50% of their villages were enrolled into the policy, and otherwise as unexposed. Means (SDs) are provided in the appendix (p 12). Means (SDs) and medians (IQRs) across the post-exposure periods are provided in the appendix (pp 12–13).

* BMI at least 28 kg/m^2^.

† Total cholesterol at least 6·22 mmol/L.

‡ December to February.

**Figure 3:**
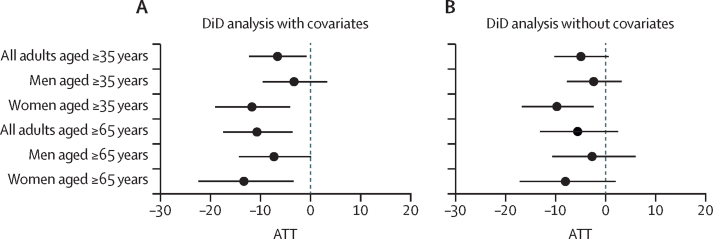
Average treatment effect on the treated of the clean heating policy on incidence of acute myocardial infarction (events per 100 000 individuals) in Beijing townships for included adults and by sex and age groups Coefficients represent the overall ATTs and 95% CIs. Dichotomous exposure variable representing townships with over 50% of villages exposed to the clean heating policy. The overall ATT estimates aggregate the dynamic group-time effects and are presented as the percent change in acute myocardial infarction incidence, calculated as (exp(coef) − 1)*100 to account for the log-transformed outcome. ATT=average treatment effect on the treated. DiD=difference in differences.

The DiD models without covariates showed consistent decreases in the incidence of acute myocardial infarction in townships included in the CHP, although the estimated effects and precision varied across sex–age groups (figure 3). In the DiD models with covariates, we estimated an overall reduction of 6·6% (95% CI –12·3 to –0·8) in the township incidence of acute myocardial infarction between the pre-exposure and post-exposure periods relative to townships not exposed to the CHP (figure 3). The distributions of the lower (2·5th) and upper (97·5th) CIs from all 6000 iterations of each DiD statistical model generally followed a normal distribution (appendix pp 10–11), indicating that our use of the median values represents their central tendencies.

We observed larger effects of the CHP on acute myocardial infarction incidence in women than men for all adults (–11·7% [95% CI –19·0 to –4·1%] *vs* –3·3% [–9·5 to 3·3]; p value for heterogeneity between men and women=0·090) and in older adults (–10·7% [–17·4 to –3·6]; p value for heterogeneity between men and women aged ≥65 years=0·30). We also observed a larger CHP effect in older men (aged ≥65 years) than in all men aged at least 35 years (–7·3% [–14·3 to 0·1] *vs* –3·3% [–9·5 to 3·3]).

**Figure 4:**
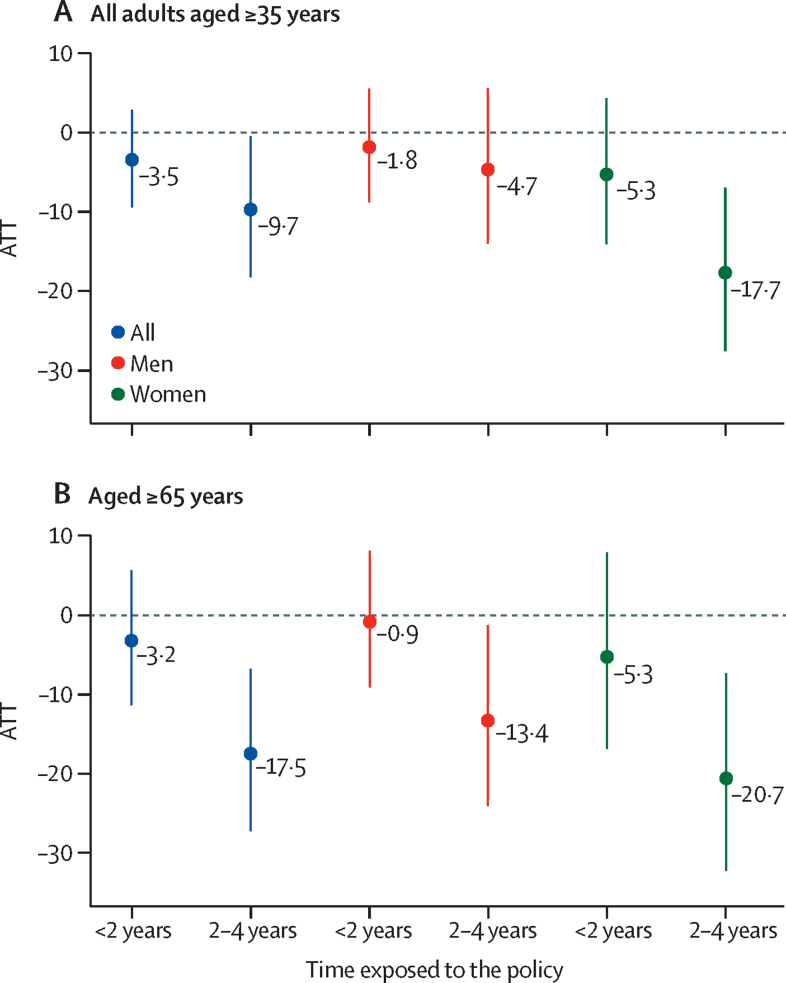
yDnamic group–time ATT of the clean heating policy on incidence of acute myocardial infarction (events per 100 000 individuals) in Beijing townships for all adults and by sex and age groups Results from covariate-adjusted difference-in-differences analyses. Dots show the ATT after less than 2 years of exposure and after 2–4 years of exposure; bars show 95% CIs. *F* statistics and p values for joint tests of equality across cohort and time ATT estimates without covariates are 12·5 and <0·0001 for all adults, 6·5 and 0·0017 for men, and 23·6 and <0·0001 for women, respectively. With covariates, the *F* statistics and p values for joint tests of equality across cohort and time ATT estimates are 9·1 and <0·0001 for all adults, 4·6 and 0·010 for men, and 15·5 and <0·0001 for women, respectively. Dichotomous exposure variable representing townships with over 50% of villages exposed to the clean heating policy. The overall ATT estimates aggregate the dynamic group-time effects and are presented as the percent change in acute myocardial infarction incidence, calculated as (exp(coef) − 1)*100 to account for the log-transformed outcome. ATT=average treatment effect on the treated.

The dynamic group–time specification showed a greater reduction in acute myocardial infarction incidence for townships exposed to the CHP for longer (figure 4), where the ATT was –3·5% (95% CI –9·5 to 2·8) for townships exposed to the CHP for less than 2 years and –9·7% (–18·3 to –0·5) for those exposed to the CHP for 2–4 years in models with covariates.

All results from our sensitivity analyses were consistent with our findings of a CHP benefit on the incidence of acute myocardial infarction in the main analysis (appendix p 15), although the magnitude of the effects and their precision differed slightly. Age-stratified analysis showed similar acute myocardial infarction effects for adults aged 65–79 years (–8·9% [95% CI –16·1 to –1·2]) and 80 years and older (–10·9% [–25·1 to 1·0]). Estimating 2-year acute myocardial infarction rates starting in November, to better temporally match the Nov 15 start of the heating season, resulted in similar magnitudes and more precise estimates for all adults (–5·7% [–10·6 to –0·6]). Using more conservative thresholds and additionally adjusting for hypercholesterolaemia resulted in smaller ATTs than in the main analysis (–8·1% [–17·9 to 2·5] and –5·1% [–10·9 to 0·8] for all adults, respectively). The use of single-year acute myocardial infarction estimates for older adults (aged ≥65 years) resulted in a small decrease in estimates and greater uncertainty compared with the main analysis (–7·3% [–18·6 to 5·3]), potentially due to greater instability in the acute myocardial infarction estimates in less-populated townships.

## Discussion

We found a reduction of 6·6% in the incidence of acute myocardial infarction in townships where over 50% of villages were participating in the CHP, indicating a potential cardiovascular benefit of the CHP averaged over the entire peri-urban and rural Beijing population. Some evidence of larger acute myocardial infarction benefits of the CHP was detected in women versus men, older adults (aged ≥65 years), and in townships exposed to the CHP for longer. Similarly, the effects of the CHP on acute myocardial infarction in adults aged 65 years or older (the age group in which two-thirds of acute myocardial infarction events occurred) was 1·6 times larger than the estimated effect of the CHP in all adults. The observed acute myocardial infarction benefits of the CHP persisted across multiple sensitivity analyses. Overall, our population-based study indicates that policies promoting clean energy transition, specifically clean heating, might provide a measurable cardiovascular benefit.

To our knowledge, this is the first study to empirically investigate the population-based health effects of northern China's ambitious and large-scale CHP, and one of few studies globally to estimate the health effects of a household energy policy.^18^ Our results are timely, as they are synchronous with ongoing and planned clean energy policies in China and other countries in a global effort to “ensure access to affordable, reliable, sustainable, and modern energy for all” (Sustainable Development Goal 7), and respond to a call-to-action from global cardiovascular societies for studies that inform targeted pollution-reducing strategies to reduce cardiovascular disease.^6^ On the basis of the observed effects of the CHP on acute myocardial infarction, we estimate that 770 (95% CI 88–1528) acute myocardial infarction events avoided can be attributed to the CHP in Beijing in 2018–19, with an additional 366 (44–682) events avoided if all eligible townships had been exposed to the CHP.

The few previous evaluations of household energy policies, mostly in high-income countries, have similarly indicated some health benefit. Residential wood-burning regulations were associated with reductions in cardiovascular hospitalisations (–7%) in California's San Joaquin Valley (USA),^19^ and with reduced cardiovascular (–17·9%) and respiratory (–22·8%) mortality in Launceston (TAS, Australia),^20^ although neither study fully controlled for secular trends in health—ie, temporal trends resulting from long-term changes in health risk profiles. Most relevant to our study are two assessments of coal-replacement policies. Small decreases in chronic lung diseases (–3·0% to –1·1%) were observed in a multi-city cohort of 8524 Chinese adults in cities where the CHP was piloted, compared with adults in cities not in the pilot. By contrast with our results, the study observed no change in physician-diagnosed cardiovascular diseases, potentially due to a post-policy period of just 1 year or confounding by other city-wide air quality and health-related policies.^21^ In a population-based study in Ireland, reductions in respiratory mortality were observed after a domestic coal heating ban, but no effect was found for cardiovascular mortality after accounting for secular changes in health.^22^ Our study provides a methodologically robust contribution to these previous studies through our controlled, before-and-after, DiD design and 4-year post-implementation assessment period.

Randomised trials of less-polluting cookstoves further support the cardiovascular benefits of transitioning from solid fuel to more efficient household stoves in our study. In Guatemala, a chimney cookstove intervention lowered exposure to air pollution and reduced the occurrence of non-specific ST-segment depression, an indicator of health conditions including myocardial ischaemia, in women (aged 38–84 years).^23^ That same intervention study and randomised trials in Nigeria and Ghana resulted in observed reductions in systolic blood pressure (–3·7 mm Hg to –1·3 mm Hg) in women assigned randomly gas, ethanol, or improved combustion biomass stoves, and are supported by non-randomised, controlled, intervention studies in Nicaragua and Bolivia (systolic blood pressure reductions ranging from –5·9 mm Hg to –5·5mm Hg).^5^ By contrast, a multi-country randomised trial did not observe a protective effect of gas stoves on gestational blood pressure,^24^ although the participants were pregnant women and younger (mean age 25 years) than in studies showing a cardiovascular benefit of intervention (mean age 28–53 years).^5^ These trials had a short duration (2–3 years) and sample sizes that were smaller than what is required to assess clinical endpoints such as acute myocardial infarction, or to assess the shorter-term versus longer-term health effects of an intervention, as we did in this study.

We were unable to investigate the mechanisms through which the CHP reduces acute myocardial infarction in this study. This heating-focused policy appears to have accelerated transition to clean energy for both heating and cooking in Beijing,^7^ which could affect well established acute myocardial infarction risk factors, such as household and outdoor air pollution and indoor temperature.^3^, ^12^ Panel studies in the Beijing region attributed a 5–6 μg/m^3^ reduction in outdoor PM_2·5_ to the CHP, but did not assess indoor air quality.^25^, ^26^ A cross-sectional study observed warmer indoor temperatures (mean difference 1·4°C) in households exposed to the CHP compared with households in nearby villages not exposed to the CHP.^10^ Household heating in winter is central to daily life, and the CHP might affect behavioural risk factors for acute myocardial infarction such as diet, physical activity, or sleep. Understanding the mechanisms through which the CHP affects health is an opportunity for future investigation.

We found consistently larger acute myocardial infarction benefits of the CHP in women. Women in China tend to spend more time indoors than men, which could result in greater benefits from a new household (indoor) stove.^27^ This difference could also be attributable to the higher rate of tobacco smoking in Chinese men (50·8%) versus women (1·9%).^28^ Continued tobacco smoking might mask the comparatively small environmental benefit of a new heater, a hypothesis supported by an observational study in China that found smaller associations between solid fuel use and cardiovascular mortality in smokers than in non-smokers.^29^ By contrast, a wood stove change-out in Launceston, Australia, reduced cardiovascular mortality in men only (–17·9% [95% CI –30·6 to –2·8]).^20^ Our rural Beijing context differs considerably from urban Australia, hindering the identification of the exact causes for the differences in findings. Behavioural factors (eg, time spent indoors or time spent near household heaters) related to exposure to the intervention could differ by gender in these two settings, and national smoking rates among men in Australia (around 10%) are much lower than those in Beijing.^30^

Our results indicate that older adults in our study might have benefited more from the CHP than younger adults, supporting previous findings in the San Joaquin Valley, where the cardiovascular benefits of a wood-burning ban were limited to adults aged 65 years and older.^19^ Older adults are more susceptible to vascular responses from changes in environmental factors because of the gradual decline in physiological processes over time and their higher prevalence of cardio-respiratory diseases.^3^, ^12^, ^19^ They are also more likely to stay at home than working-age adults,^27^ which could affect their duration of exposure to an indoor intervention.

Reductions in acute myocardial infarction incidence were larger in townships exposed to the CHP for longer in our study, which could be influenced by several factors. Households might require time to adapt to using the new heaters or possibly finish burning any stored solid fuel, and thus only later experience the full benefits of intervention. Exposure to PM_2·5_ over a timespan of years increases cardiovascular risk by a larger magnitude than exposure over days or months, suggesting that the acute myocardial infarction benefits of an air pollution policy might accrue over time.^3^ Also, some observed group–time effects might be an artifact of our township-level CHP variable, where the percentage of exposed villages in a township can only increase over time. Among the cohort of townships considered exposed to the CHP by 2016–17 in our study, we observed a 5% average increase in the proportion of villages with the CHP in 2018–19. Additionally, use of 2-year periods meant that many townships considered exposed to the CHP in 2016–17 were not assigned into the policy for the entire first post-exposure period, but were so for the second period. This error in exposure measurement potentially biases our estimates towards the null more for the first post-exposure period than the second.

Our DiD approach was subject to several assumptions. We assumed that anticipation of the CHP did not differ between exposure groups. It was generally known that the CHP would start in areas with updated electricity grids near the urban core and gradually expand into more remote areas of Beijing. Outside of those geographical parameters, some villages were directly assigned to the CHP whereas others applied and were selected by the local government, but villages were generally unaware whether they would be selected for the CHP.^10^ We also cannot entirely rule out the possibility that other policies differentially affected acute myocardial infarction by exposure group, which could lead to over-estimation or under-estimation of the effects. Beijing implemented many rural development and air quality measures (eg, traffic restrictions and retiring coal-burning power plants) that could potentially affect health.^31^ To our knowledge, none of these measures followed the same spatiotemporal patterns of the CHP or would have differentially affected villages included in the CHP versus those not included. Major motor vehicle restrictions and coal-burning boiler replacements were implemented before the CHP, and thus should not affect our results. The proportion of townships near retired coal-fired power plants in the post-exposure period was slightly higher in townships exposed to the CHP than townships not exposed, and we were able to adjust for exposure to a retired plant in our analysis. We excluded urban townships with the assumption that most of their villages had central heating and were ineligible for the CHP. Urban-core townships are also more likely to be affected by traffic-related policies, further justifying their exclusion. Finally, our analysis assumes that, in the absence of the CHP, the trends in acute myocardial infarction incidence in townships exposed to the CHP versus not would have remained the same over time. Although we cannot verify this assumption, we observed similar trends in acute myocardial infarction incidence across exposure groups in the 8 years before the CHP and did our analysis using a combination of regression adjustment and inverse probability weights, all of which improves the credibility of this assumption.

Strengths of our study include the population-based assessment of a large-scale household energy policy using a quasi-experimental design that controlled for secular trends in health and important covariates. We estimated acute myocardial infarction incidence from both hospitalised cases and out-of-hospital deaths obtained from a high-quality surveillance system covering all of Beijing. Our sensitivity analyses demonstrated that our findings remained consistent across many different analytical decisions. But this study also has several limitations to consider when interpreting our results. First, we defined exposure to a village-level policy at the township level to match the available spatial resolution of acute myocardial infarction data. Townships considered exposed to the CHP in our study thus included villages that were not in the policy and vice versa, which is most likely to be non-differential misclassification and would usually bias estimates towards the null. Applying a more conservative definition of exposure to the CHP in a sensitivity analysis produced larger ATTs. Second, the outcome of interest in this study (township incidence rate of acute myocardial infarction) was estimated with assumptions about its spatial structure that could potentially bias the estimates of acute myocardial infarction in either direction. However, any resulting error is not likely related to implementation of the CHP and would thus most likely decrease our precision in estimating the effects of the CHP on acute myocardial infarction. Future studies might consider individual-level analyses to reduce error in both CHP exposure and outcome assessments. Third, although our township-level CHP exposure variable might capture some village-level spillover effects of the CHP (eg, improved outdoor air quality), such effects remain possible and would likely bias our results towards the null. Finally, our main analysis defined exposure to the CHP on the basis of the proportion of villages assigned into the CHP by the second year of each 2-year calendar period, meaning that some acute myocardial infarction cases could have occurred before villages installed their CHP-subsidised heat pumps, which would most likely bias our results towards the null. Estimating acute myocardial infarction from November to October rather than calendar year to better temporally align with the start of the heating season in November showed similar overall ATTs. Among older adults, single-year acute myocardial infarction estimation still showed an effect of the CHP on acute myocardial infarction but with smaller estimates, potentially due to the greater instability of the acute myocardial infarction estimates in less-populated townships.

Our population-based study provides some of the first empirical evidence of cardiovascular benefits attributable to a household energy policy. China has shown an exceptional capacity to implement clean energy policies that provide more efficient household stoves and fuel to hundreds of millions of households^32^ at a cost and scale that could be challenging to replicate in many settings. Still, the results of our study, combined with those of several trials of clean cookstoves and many observational studies of air pollution and cardiovascular outcomes, indicate that even smaller-scale interventions might provide a health benefit, and should thus motivate the continued investment in clean household energy in China and other countries that rely on solid fuel for household heating and cooking.

### Contributors

### Data sharing

The individual patient data used for this study were obtained from the Beijing Municipal Health Big Data and Policy Research Center and cannot be shared publicly given institutional regulations and data confidentiality agreements. However, data may be requested by researchers from the above data holder authorities for research purposes. The analytical methods can be reproduced based on the details provided in this Article, and the statistical code is available on GitHub (https://github.com/mlee725/Code-for-difference-in-differences-analysis-for-policy-effect-on-AMI-.git).

## Declaration of interests

We declare no competing interests.
