# Supplementary material for: Effects of a coal to clean heating policy on acute myocardial infarction in Beijing: a difference-in-differences analysis
Source: Lancet Planet Health. 2024 Nov 5;8(11):e924–32. doi: 10.1016/S2542-5196(24)00243-2 (PMC11540976; doi:10.1016/S2542-5196(24)00243-2)
Supplement: Supplementary appendix [file mmc1.pdf]

THE LANCET
Planetary Health

Supplementary appendix

This appendix formed part of the original submission and has been peer reviewed. We post it as supplied by the authors.

Supplement to: Lee M, Chang J, Deng Q, et al. Effects of a coal to clean heating policy on acute myocardial infarction in Beijing: a difference-in-differences analysis. *Lancet Planet Health* 2024; **8:** e924–32.

Supplementary appendix

Supplement to: Effects of a Coal-to-Clean Heating Policy on Acute Myocardial Infarction in Beijing: A Difference-in-Differences Analysis

Table of Contents

Supplement 1: The Clean Heating Policy (CHP) and its implementation in northern China 2

Supplement 2: Determining township eligibility for the Clean Heating Policy (CHP) 2

Supplement 3: Geolocating villages in Beijing by Clean Heating Policy (CHP) exposure status 2

Supplement 4: Registration of acute myocardial infarction (AMI) hospital admissions and out-of-hospital deaths 2

Supplement 5: Age-standardization and modeling of township acute myocardial infarction (AMI) incidence for Beijing 3

Supplement 6: Covariate descriptions and data sources 4

Supplement 7: Multiple time point difference-in-differences (DiD) methodological details 5

Figure S1. Proportion of villages in Beijing townships participating in the Clean Heating Policy (CHP) by (A) December 31, 2017 and (B) December 31, 2019 6

Figure S2. Temporal trends in mean and median of log-transformed incidence of acute myocardial infarction (AMI) among adults in eligible Beijing townships from 2007 to 2019, by Clean Heating Policy (CHP) exposure group 7

Figure S3. Temporal trends in median age-standardized incidence of acute myocardial infarction (AMI) for eligible Beijing townships by sex-age group and Clean Heating Policy (CHP) exposure group for (A) all adults and (B) adults ages 65y+ 8

Figure S4: Temporal trends in selected covariates in Beijing study townships by Clean Heating Policy (CHP) exposure group 9

Figure S5: Histograms of all 6000 lower 95% CI estimates of the average treatment effect on the treated (ATT). Estimates were generated by running each difference-in-differences analysis 6000 times using the 6000 random draws generated from the posterior distributions of the Bayesian model for acute myocardial infarction (AMI) estimation 10

Figure S6: Histograms of all 6000 upper 95% CI estimates of the average treatment effect on the treated (ATT). Estimates were generated by running each difference-in-differences analysis 6000 times using the 6000 random draws generated from the posterior distributions of the Bayesian model for acute myocardial infarction (AMI) estimation 11

Table S1: Township characteristics (mean and [standard deviation, SD]) over time and by Clean Heating Policy (CHP) exposure group 12

Table S2: Township characteristics (median [interquartile range, IQR]) over time and by Clean Heating Policy (CHP) exposure group 13

Table S3: Average treatment effect on the treated (ATT) of the Clean Heating Policy (CHP) on acute myocardial infarction (AMI) incidence Beijing townships: Results from multiple sensitivity analyses. The ATTs are presented as the percent change in AMI events per 100,000 population with 95% confidence intervals in brackets 15

Supplement 1: The Clean Heating Policy (CHP) and its implementation in northern China

The Clean Heating Policy (CHP) was part of the large national effort to improve air quality in northern China with the aim of transitioning villages from residual coal burning for heating to 'clean' electric or gas-powered heating. This initiative is referred to by many names including the 'northern region clean winter heating plan" (北方地区冬季清洁取暖规划) and "clean energy for rural heating" (农村采暖用能清洁化) in government documents. Within the peer-reviewed literature it has also been referred to as the coal-to-gas/electricity policy,[1] the coal ban,[2] residential coal-switch policy,[3] coal-to-clean energy policy,[4] and clean heating project.[5]

In Beijing, it was generally understood that the policy would first be implemented in the plains areas with updated electric grids and then gradually expand into more remote and mountainous areas of Beijing. Beyond this, there were – to the best of our knowledge and based on the scientific literature, policy documents, and discussion with leaders of local villages enrolled in the policy – no other discernible factors that influenced village enrollment into the policy, to the extent that we consider village assignment into the policy as plausibly random. Villages did not know if or when they would be enrolled, as most were required to enter the policy whereas others applied and were selected.[2] The brand of heat pump and fuel source (e.g., electricity or gas) was determined by local infrastructure and our data on policy implementation indicate that most (81%; n = 2509) villages selected to implement electric heat pumps. Village enrollment into the policy in Beijing continued past the end of our study period, however we were unable to incorporate additional years into our analysis due to the availability of health data only through 2019.

Supplement 2: Determining township eligibility for the Clean Heating Policy (CHP)

Villages connected to district heating systems that supply heat from central locations were ineligible for the CHP. We did not have access to village-level data on centralized heating, and therefore needed to identify townships in which most residents were likely already connected to centralized heating and thus ineligible for the policy. We excluded 151 townships in which the majority of their villages had an urban designation (She Qu, 社区) which are usually connected to centralized heating. We additionally excluded a township with an urban designation in its name (Jie Dao, 街道) rather than Zhen (镇) or Xiang (乡) and further excluded four townships in the urban core with less than 10% of villages enrolled in the policy, indicating that the vast majority of villages in those townships had centralized heating. Prior to categorizing a township as ineligible, we also visually assessed satellite images of the township for urban build up (e.g., high rise apartment buildings) which indicates access to centralized heating. In total, 156 of the 307 townships were considered ineligible and excluded from the analysis, resulting in a final sample size of 151 townships.

Supplement 3: Geolocating villages in Beijing by Clean Heating Policy (CHP) exposure status

After excluding several duplicate village entries (n=25), the remaining 2519 villages listed as participating in the CHP between 2015 and 2019 were matched with villages in a complete administrative dataset obtained from the Chinese National Bureau of Statistics in 2019 (n=7168).[6] We geo-located villages using their village, township, and district names while considering similar names or alternative spellings in the same township or a neighboring township and by also searching Baidu Maps[7] and Baidu Baike.[8] Ten of the 2519 villages listed as exposed to the policy were excluded from the dataset either because they could not be geolocated (n=5) or were no longer independent administrative villages by 2019 (n=5) according to the National Bureau of Statistics.[6] Nineteen villages enrolled in the policy were not originally listed in the 2019 administrative villages dataset and were added after geolocating them on Baidu Maps[7] and verify their existence on Baidu Baike.[8] The final village dataset contained 7187 villages, of which 2509 were exposed to the policy by December, 2019. Most villages were listed as entering the policy in a single year, though others entered over two (n=718), three (n=141), or four (n=5) years and were considered as exposed to the policy in the first year of entry.

Supplement 4: Registration of acute myocardial infarction (AMI) hospital admissions and out-of-hospital deaths

Acute myocardial infarction (AMI) events and deaths were extracted from the Beijing Cardiovascular Disease Surveillance System, which records all secondary- and tertiary-level hospital admissions and out-of-hospital deaths. The surveillance system does not include primary hospitals which provide basic medical, preventative, and rehabilitation services. Most AMI patients go directly to secondary or tertiary hospitals[9] and those presenting at a primary hospital are referred to a secondary or tertiary hospital for medical treatment. Incidence of hospital-diagnosed AMI was obtained from the hospital of diagnosis. Information on patient residence was collected at the hospital, which was then used to assign their township of residence for our study.

Supplement 5: Age-standardization and modeling of township acute myocardial infarction (AMI) incidence for Beijing

Age- and sex-standardized incidence rates of AMI (events per 100,000 population) were first calculated by direct standardization using the Beijing population from the 2010 national population census as the reference. For the main analysis, age and sex categories for standardization included men and women aged 35 to 64 and ≥65 years with corresponding weights of 43% and 8% for men, and 40% and 9% for women, respectively. As a sensitivity analysis, we age-standardized using more resolved age groups: 35-49y, 50-64y, 65-79y, 80y+ with weights of 50%, 33%, 14%, and 3%, respectively. In the sensitivity analysis with adults 65-79y and 80y+, we used the corresponding weights of 82% and 18%, respectively.

We selected the Besag-York-Mollié (BYM) model[10] to estimate township AMI because it takes into account that AMI data may be spatially correlated and observations in neighbouring townships may be more similar than observations in townships that are farther away. Specifically, the BYM model combines an intrinsic conditional autoregressive (ICAR) component, which captures spatial dependence by borrowing strength from neighbouring areas, with an independent random effect that accounts for variations not explained by the spatial structure. This dual component framework is frequently implemented for disease mapping to produce smoothed estimates at the small-area level that are less impacted by the oversized effect that random fluctuations in AMI cases can have in small populations, particularly when data are sparse or unevenly distributed across spatial units. The BYM model can effectively manage issues such as overdispersion and spatial clustering, making it an ideal choice for spatial estimation of health outcomes like AMI where understanding the spatial patterns and variability at a granular level was the goal. The model was expressed as follows:

$$Events_d \sim Poisson(m_d * Population_d)$$
$$log(m_d) = \alpha_0 + b_d + h_d$$

where $Events_d$ is the number of reported AMI events in township d, $Population_d$ is the population size of township d, and m_d is the AMI incidence for township d and for each time period-sex-age group. The α_o is the common intercept, i.e., overall AMI incidence. The terms b and h are components of the BYM model and described in detail elsewhere.[11] Briefly, the random effects denoted by b_d were assumed to follow intrinsic conditional autoregressive distributions that allowed for smoothing over adjacent townships' rates. The random effects denoted by h_d were assumed to follow independent mean zero normal distributions.

In the BYM model, the estimated AMI incidence values for each township were influenced by each township's own data and by neighbouring townships through spatially structured random effects with conditional autoregressive prior distribution as well as globally through a spatially unstructured normal prior distribution. In this specification, the weighting matrix W is symmetric ($w_{d,d'} = w_{d',d}$) but not reflective ($w_{d,d} = 0$) with a township not able to be its own neighbour and $w_{d,d'}$ is nonnegative. We assumed a first-order neighborhood structure (i.e. binary, first-order, adjacency matrix) where $w_{d,d'}$=1 for geographically adjacent townships and $w_{d,d'}$=0 otherwise. The township's spatial random effect b_d is normally distributed and with a mean based on the average of the neighboring townships.

The variance is inversely related to the number of neighbors. The spatial structure of the model was expressed as:

$$b_d | b_{d'}, d' \in \partial_d \sim N\left(\frac{1}{N_d}\sum_{d'\in\partial_d} b_{d'}, \frac{\tau^2}{N_d}\right)$$

where ∂_d is the set index containing the neighboring townships d, N_d is the number of townships, and τ^2 is the unknown variance.

Using this approach, we assume that 'neighbors' are all townships with shared geographic boundaries and that all neighbors have equal influence on a given township's estimate of AMI. We chose a small-area estimation approach that derives weights based on distance between township centroids because few townships in Beijing have atypical (e.g., L shaped) spatial patterning also because we did not have a clear rationale for considering other spatial structures, such as a centroid approach, which only adds assumptions about the spatial structure. For example, in rare cases of L shaped patterning, the centroid may be far away from a neighbouring township boarder but the population might live close to the border, and thus have AMI outcomes that more similar.

We visually assessed convergence using trace plots. We ran two chains, discarding the first 12000 and thinning the remainder by 100 to obtain 6000 post-burn-in draws from the posterior distribution of model parameters. We reported the posterior mean of these 6000 draws.

Supplement 6: Covariate descriptions and data sources

Covariates	Definition	Spatial resolution	Years with data available	Source
Secondary school education attainment (%)	Proportion population with secondary school education or higher divided by the population ages 6y and older	Township	2010	2010 national population census[12]
Employment in agriculture (%)	Proportion population working in agriculture and related industries (excluding those working in agricultural services) divided by total population			
Unemployment (%)	Proportion population ages 16y and older who are not employed but able to and seeking work divided by the economically active population (i.e., population 16y and older who are able and wanting to work)	District		
Smoking (%)	Proportion population reporting to currently smoke tobacco	District	2014 and 2017	Beijing Chronic Disease and Risk Factors Surveillance
Obesity (%)	Proportion population with body mass index (BMI) $\geq 28 kg/m^2$			
Hypercholesterolemia (%)	Proportion population total cholesterol ≥6·22 mmol/L			
Exposure to a retired coal-fired power plant	Dichotomous variable where a township was considered exposed if it's centroid was within a 20 km radius from a power plant that was retired during the study period and otherwise as unexposed. The radius of <20 km is based on Chen et al. (2020)[13]	Township	2013-2019	Global Energy Monitor[14]
Health care access	Defined as the number of hospital beds per 1000 population. Health care access was measured using an enhanced two-step floating catchment area method based on a Gaussian function. This method incorporates the interaction between health care supply and potential demand. The number of hospital beds was set as the health care supply capacity. The demand size refers to the size of population in the hospital catchment area.	Township	2013-2019	Chang et al. (2023)[15]
Variability in daily annual outdoor temperature (°C)	Standard deviation of the distribution of daily outdoor temperature calculated across all grid cells (0·25° x 0·25°) within the polygon delineating each township. We used the standard deviation as evidence from Beijing indicates that the variability in outdoor temperature is more strongly associated with risk of AMI than the average temperature.[16,17]	Township	2013-2019	ERA5 hourly data[18]
Variability in daily heating season outdoor temperature (°C)	Standard deviation of the distribution of daily outdoor temperature calculated for the months of December, January, and February across all grid cells (0·25° x 0·25°) within the polygon delineating each township.			

Supplement 7: Multiple time point difference-in-difference (DiD) methodological details

Our selected DiD approach assumes that, in the absence of participating in the CHP, the counterfactual path of AMI incidence in CHP-exposed townships would be the same as the path of AMI incidence experienced by townships not exposed (i.e., control) to the policy (i.e. the assumption of parallel trends). Simple comparisons of exposed and unexposed townships after CHP implementation are likely to be biased by unmeasured characteristics that are associated with risk of AMI (e.g., township socioeconomic status, access to health-promoting services). Similarly, comparisons of only CHP-exposed townships before and after the policy are susceptible to bias by other factors associated with changes in the population risk profiles for AMI over time (i.e., secular trends). Thus, by comparing the changes in AMI among CHP-exposed townships to the changes in AMI among townships not yet exposed, the DiD approach controls for unmeasured time-invariant characteristics of townships as well as for secular trends affecting outcomes in all townships that are unrelated to the policy.

We utilised the DiD identification strategy introduced by Callaway and Sant'Anna (2021),[19] which allows for heterogeneity in the policy's effect over time. More specifically, it allows the effect of participating in the policy to vary across time periods or across duration of exposure to the policy since there could be a behavioural updating period as villages have more time to adapt to the new policy. It is also possible that the duration of behavioural updating could change later in the policy as more villages are exposed and the policy is better recognized and understood by participating villages.

Using the did R package from Callaway & Sant'Anna,[20] we used a difference-in-differences with multiple time periods approach to estimate the average treatment effect on the treated (ATT) for each CHP-exposed (treated) group *g* at each time point *t* [i.e., ATT (g,t)], where the exposure 'groups' or 'cohorts' are defined based on when townships were first considered exposed to the policy and using not-yet-exposed townships as controls. The group-time specific estimates are derived in the regression by subsetting the observations at each group and time period covering pre- and post-intervention, using the following linear regression specification:[19, 21]

$$y_{it} = \beta_0^{gt} + \beta_1^{gt} G + \beta_2^{gt} T + \beta_3^{gt} (G * T) + \varepsilon^{gt}$$

where y_{it} is the number of AMI events per 100,000 population for township *i* at time point *t,* G and T are the indicator variables for group and time, respectively, and β_3^{gt} is the average treatment effect on the treated for each group at a given time period [ATT(g,t)]. In the above equation, the group-time average treatment effect, ATT(g,t) or β_3^{gt}, can be expressed as:

$$ATT(g,t) = E[Y_t(g) - Y_t(0) | G_g = 1]$$

where $Y_t(g)$ and $Y_t(0)$ are the potential number of AMI events per 100,000 population for a township at a given time point *t* with treatment as part of group *g* and without exposure to the policy, respectively. This approach effectively creates township and time fixed effects by utilising within-township change and estimating each time point separately.

Next, we used event study (dynamic) aggregation to summarise the group-time average treatment effects, $ATT(g,t)$, as described in Callaway and Sant'Anna.[19] Briefly, this approach uses a weighted-average of the ATT(g,t) estimates grouped by length of time since exposure to the policy, using the following specification:

$$\theta_D(e) = \sum_{g \in G} 1\{g + e \leq T\} P(G = g | G + e \leq T) ATT(g, g + e)$$

where θ_D is the ATT for a given time period (e), i.e. $e = t - g$. The overall treatment effect, θ_D^o, was denoted as:

$$\theta_D^o = \frac{1}{T-1} \sum_{e=0}^{T-2} \theta_D(e)$$

Figure S1: Proportion of villages in Beijing townships participating in the Clean Heating Policy (CHP) by (A) December 31, 2017 and (B) December 31, 2019

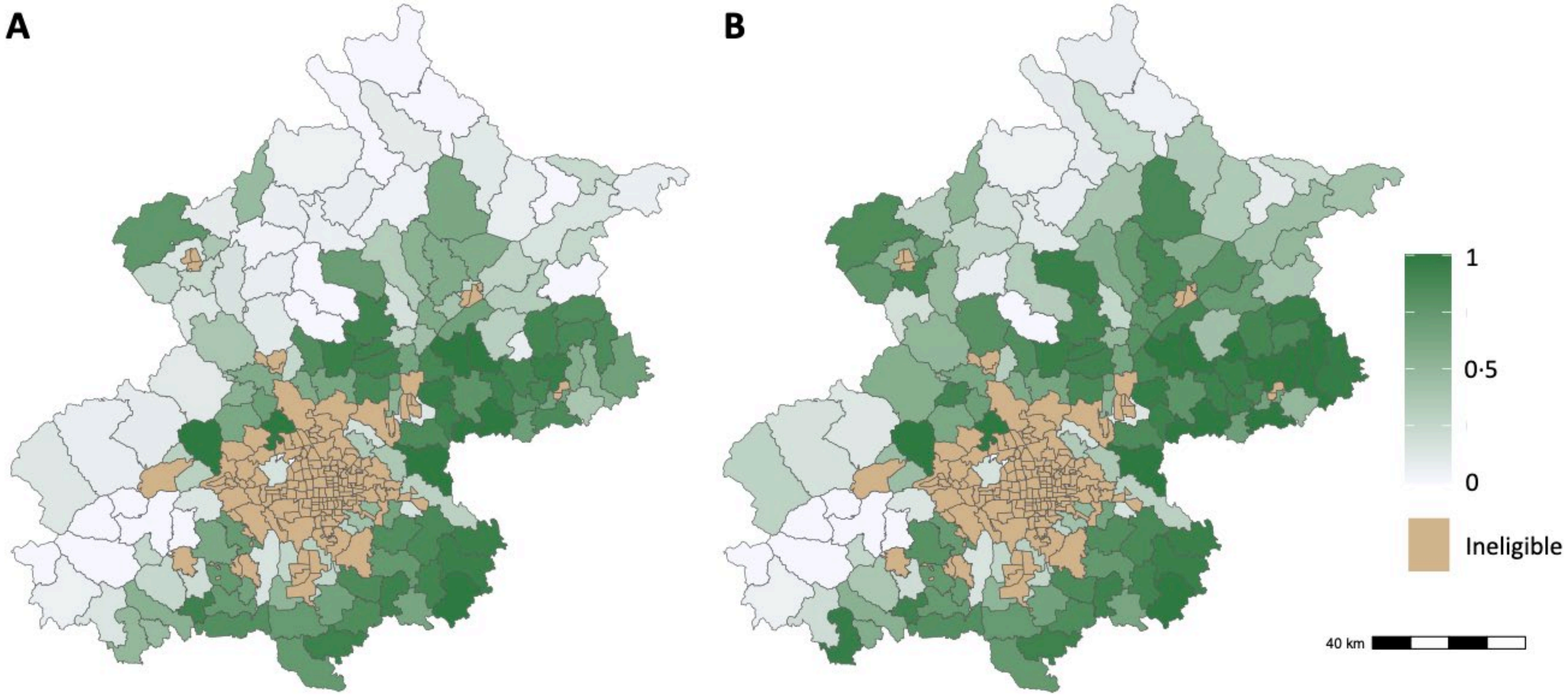

Notes: Townships comprised of mostly urban villages connected to central heating were considered 'ineligible' for the policy (shaded in light brown) and thus excluded from the analysis.

Figure S2: Temporal trends in mean and median of log-transformed incidence of acute myocardial infarction (AMI) among adults in eligible Beijing townships from 2007 to 2019, by Clean Heating Policy (CHP) exposure group.

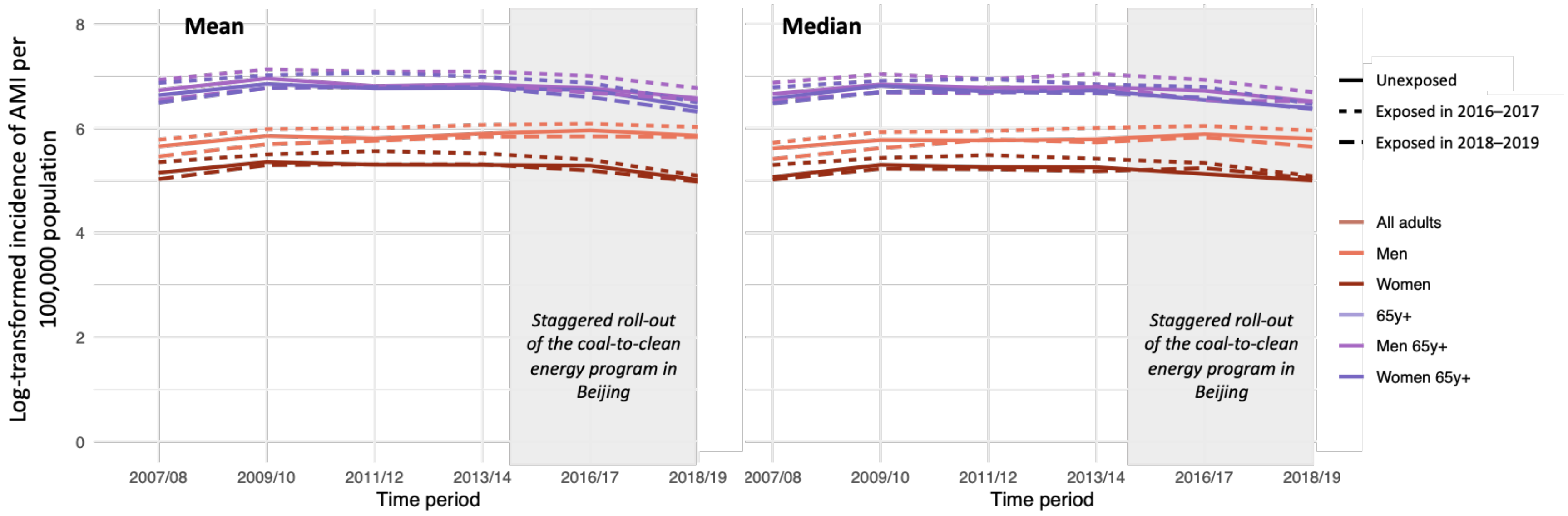

P-values for joint tests that all pre-policy trends equal 0 without covariates are 0·31 for all adults, 0·17 for men, and 0·53 for women, respectively. With covariates, the p-values are 0·87 for all adults, 0·86 for men, and 0·95 for women, respectively.

Notes: Eligible townships were considered "exposed" to the CHP if over 50% of their villages were participating in the policy. *Unexposed* refers to the group of townships that were not exposed to the CHP throughout the study period. *Exposed in 2016-2017* refers to the group of townships that were first exposed to the CHP in 2016-2017. *Exposed in 2018-2019* refers to the group of townships that considered unexposed in 2016-2017 and first exposed to the CHP in 2018-2019.

Figure S3: Temporal trends in median age-standardized incidence of acute myocardial infarction (AMI) for eligible Beijing townships by sex-age group and Clean Heating Policy (CHP)-exposure group for (A) all adults and (B) adults ages 65y+

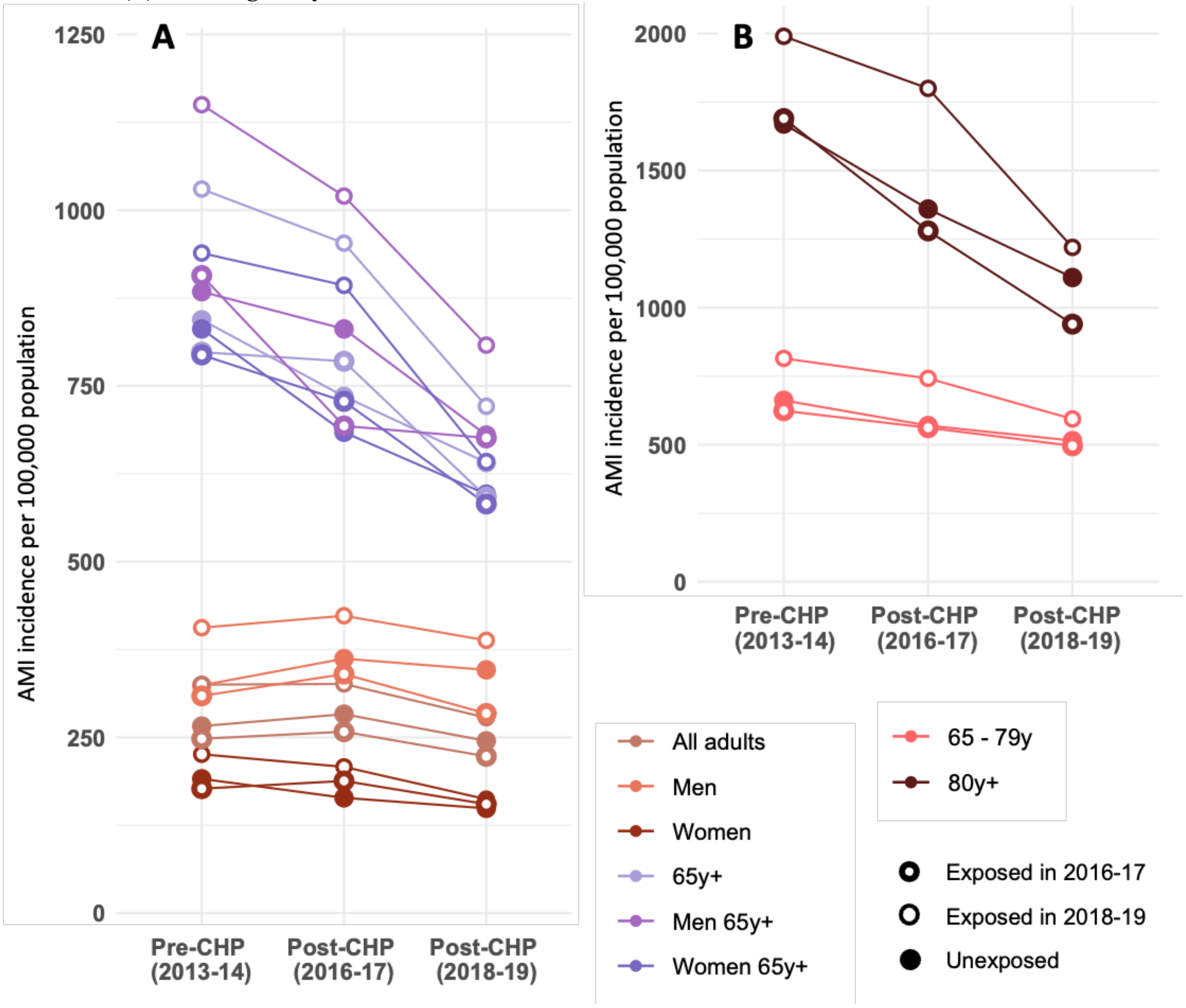

Notes: Eligible townships were considered "exposed" to the CHP if over 50% of their villages were participating in the policy. *Unexposed* refers to the group of townships that were not exposed to the CHP throughout the study period. *Exposed in 2016-2017* refers to the group of townships that were first exposed to the CHP in 2016-2017. *Exposed in 2018-2019* refers to the group of townships that considered unexposed in 2016-2017 and first exposed to the CHP in 2018-2019.

Figure S4: Temporal trends in selected covariates in Beijing study townships by Clean Heating Policy (CHP) exposure group

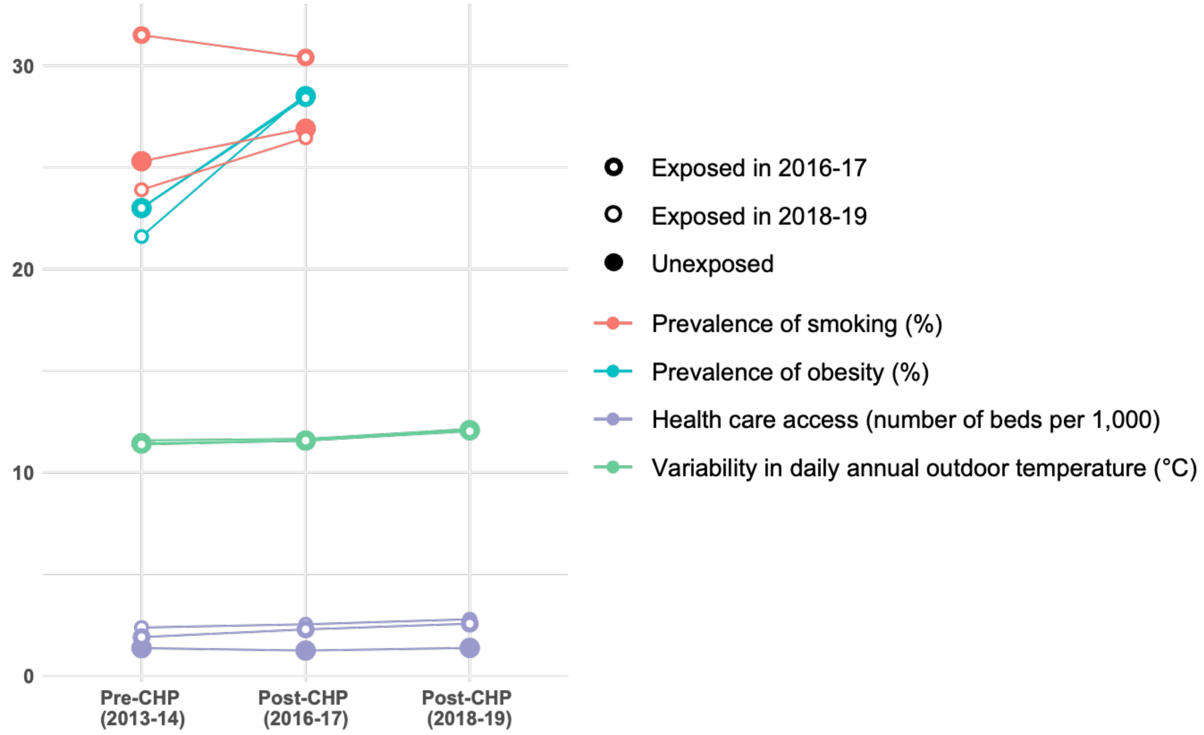

Notes: Eligible townships were considered as "exposed" to the CHP if over 50% of their villages were participating in the policy. *Unexposed* refers to the group of townships that were not exposed to the CHP throughout the study period. *Exposed in 2016-2017* refers to the group of townships that were first exposed to the CHP in 2016-2017. *Exposed in 2018-2019* refers to the group of townships that considered unexposed in 2016-2017 and first exposed to the CHP in 2018-2019. Health care access was defined as the number of hospital beds per 1000 population. Variability in annual outdoor temperature was calculated as the standard deviation of the distribution of daily outdoor temperature. Prevalence data for obesity and smoking were not available for the 2018-2019 post-CHP period.

Figure S5: Histograms of all 6000 lower 95% CI estimates of the average treatment effect on the treated (ATT). Estimates were generated by running each difference-in-differences analysis 6000 times using the 6000 random draws generated from the posterior distributions of the Bayesian model for acute myocardial infarction (AMI) estimation

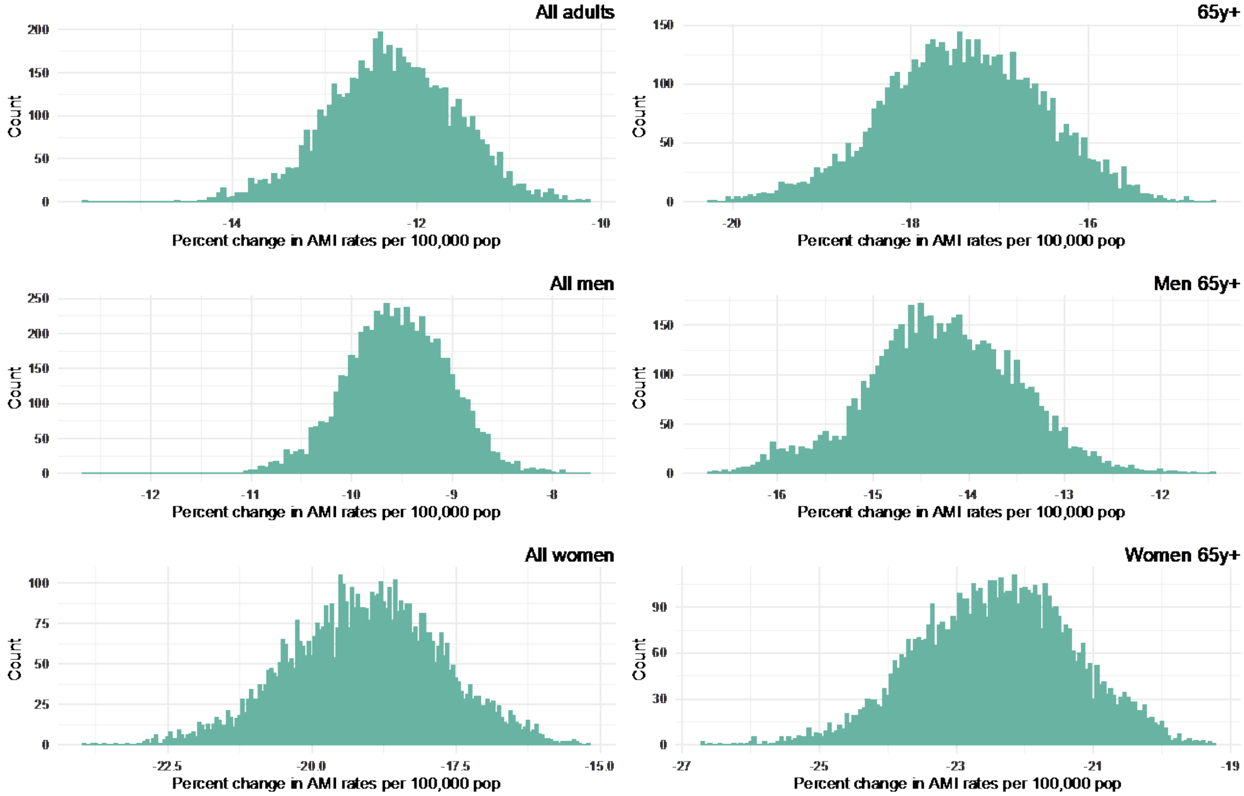

Figure S6: Histograms of all 6000 upper 95% CI estimates of the average treatment effect on the treated (ATT). Estimates were generated by running each difference-in-differences analysis 6000 times using the 6000 random draws generated from the posterior distributions of the Bayesian model for acute myocardial infarction (AMI) estimation

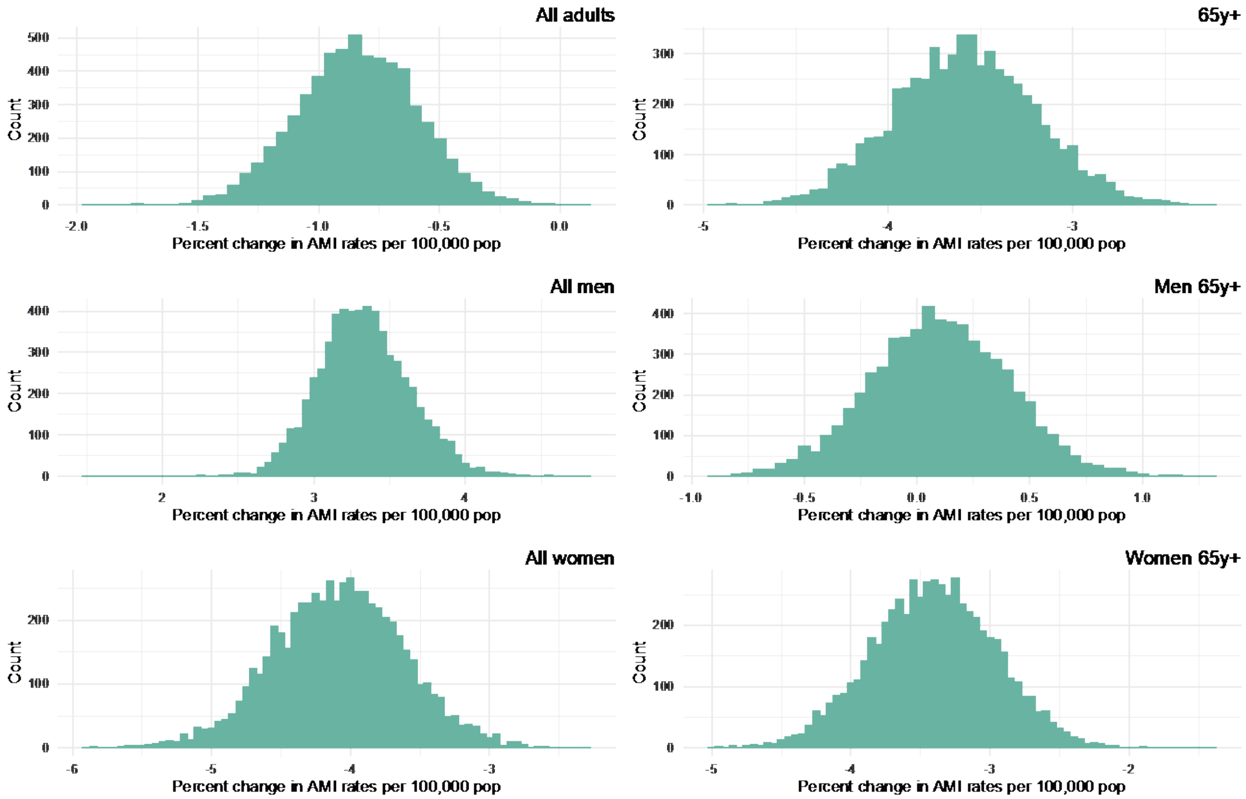

Table S1: Township characteristics (mean and [standard deviation, SD]) over time and by Clean Heating Policy (CHP) exposure group

Characteristic	Unexposed (n=59)	Exposed in 2016-17 (n=75)	Exposed in 2018-19 (n=17)	Unexposed (n=59)	Exposed in 2016-17 (n=75)	Exposed in 2018-19 (n=17)	Unexposed (n=59)	Exposed in 2016-17 (n=75)	Exposed in 2018-19 (n=17)
	Pre-CHP (2013-14)			Post-CHP (2016-17)			Post-CHP (2018-19)		
Incidence of acute myocardial infarction (events per 100,000 population)									
All adults	286 [89]	344 [97]	276 [98]	297 [91]	335 [90]	266 [84]	254 [64]	292 [107]	247 [72]
Men	366 [130]	432 [125]	344 [109]	390 [121]	441 [115]	347 [112]	352 [108]	415 [176]	343 [116]
Women	201 [60]	250 [80]	203 [92]	198 [77]	222 [72]	180 [62]	150 [36]	163 [43]	146 [33]
Adults 65y+	904 [278]	1140 [381]	909 [339]	858 [326]	1030 [350]	765 [288]	658 [169]	762 [254]	623 [169]
Men	939 [296]	1200 [392]	931 [298]	875 [309]	1110 [364]	800 [304]	720 [213]	873 [308]	699 [236]
Women	872 [297]	1090 [406]	889 [411]	843 [378]	967 [367]	734 [294]	603 [181]	663 [226]	556 [145]
Population working in agriculture (%)	76% [17]	76% [10]	72% [15]	76% [17]	76% [10]	72% [15]	76% [17]	76% [10]	72% [15]
Population unemployed (%)	5% [2]	4% [1]	4% [1]	5% [2]	4% [1]	4% [1]	5% [2]	4% [1]	4% [1]
Population with secondary school education or higher (%)	26% [10]	29% [7]	34% [12]	26% [10]	29% [7]	34% [12]	26% [10]	29% [7]	34% [12]
Population currently smoking (%)	30% [9]	26% [7]	31% [11]	27% [5]	27% [5]	30% [5]	27% [5]	27% [5]	30% [5]
Population with obesity (%)	24% [5]	22% [5]	23% [4]	28% [3]	28% [2]	28% [2]	28% [3]	28% [2]	28% [2]
Population with hypercholesterolemia (%)	7% [3]	7% [2]	7% [2]	8% [3]	8% [3]	7% [4]	8% [3]	8% [3]	7% [4]
Exposure to a retired coal-fired power plant (% of townships)	15%	9%	6%	31%	21%	6%	31%	21%	6%
Health care access (number of hospital beds per 1,000 population)	1·9 [1·8]	2·5 [1·0]	2·2 [1·2]	2·0 [2·1]	2·6 [1·2]	2·4 [1·3]	2·4 [2·5]	3·15 [1·5]	3·31 [2·0]
Variability in daily outdoor temperature (°C, annual)	11·4 [0·2]	11·6 [0·1]	11·4 [0·2]	11·5 [0·2]	11·6 [0·1]	11.5 [0.1]	12·0 [0·2]	12·1 [0·1]	12·0 [0·2]
Variability in daily outdoor temperature (°C, heating season)	3·3 [0·2]	3·1 [0·1]	3·3 [0·2]	3·6 [0·3]	3·3 [0·2]	3.6 [0.2]	3·8 [0·4]	3·6 [0·3]	3·9 [0·2]

Notes: Eligible townships were considered as "exposed" to the CHP if over 50% of their villages were participating in the policy. *Unexposed* refers to the group of townships that were not exposed to the CHP throughout the study period. *Exposed in 2016-2017* refers to the group of townships that were first exposed to the CHP in 2016-2017. *Exposed in 2018-2019* refers to the group of townships that considered unexposed in 2016-2017 and first exposed to the CHP in 2018-2019. Heating season refers to the months of December, January, and February. Obesity was defined as having a BMI ≥28kg/m^2. Hypercholesterolemia was defined as

total cholesterol ≥6·22 mmol/L. Townships were considered “exposed” to retired coal-fired power plants if they were within a 20km radius of a closed plant, and otherwise as “unexposed”.

Table S2: Township characteristics (median [interquartile range, IQR]) over time and by Clean Heating Policy (CHP) exposure group

Characteristic	Unexposed (n=59)	Exposed in 2016-17 (n=75)	Exposed in 2018-19 (n=17)	Unexposed (n=59)	Exposed in 2016-17 (n=75)	Exposed in 2018-19 (n=17)	Unexposed (n=59)	Exposed in 2016-17 (n=75)	Exposed in 2018-19 (n=17)
	**Pre-CHP (2013-14)**			**Post-CHP (2016-17)**			**Post-CHP (2018-19)**		
Incidence of acute myocardial infarction (events per 100,000 population)									
All adults	266 [225–350]	325 [277–383]	248 [224–286]	281 [234–339]	326 [269–373]	258 [216–312]	236 [211–298]	278 [252–310]	223 [194–290]
Men	327 [280–439]	406 [356–487]	309 [291–380]	361 [310–450]	423 [357–499]	340 [272–389]	330 [274–420]	388 [348–419]	284 [262–422]
Women	192 [161–247]	226 [198–283]	177 [158–223]	168 [149–232]	208 [176–258]	188 [138–207]	149 [123–174]	162 [134–182]	155 [125–167]
Adults 65y+	854 [694–1064]	1034 [878–1267]	798 [728–1015]	737 [635–997]	953 [803–1196]	785 [554–896]	641 [532–765]	721 [617–824]	592 [499–717]
Men	890 [773–1053]	1147 [927–1382]	907 [673–1026]	831 [657–1007]	1023 [846–1260]	693 [611–1009]	678 [564–836]	808 [740–946]	676 [570–785]
Women	836 [641–1086]	939 [829–1262]	794 [652–1006]	695 [593–997]	893 [726–1140]	728 [515–919]	596 [455–714]	642 [512–776]	582 [425–650]
Population working in agriculture (%)	81% [70–88]	78% [73–83]	79% [65–82]	81% [70–88]	78% [73–83]	79% [65–82]	81% [70–88]	78% [73–83]	79% [65–82]
Population unemployed (%)	4% [4–6]	4% [4–4]	4% [4–4]	4% [4–6]	4% [4–4]	4% [4–4]	4% [4–6]	4% [4–4]	4% [4–4]
Population with secondary school education or higher (%)	24% [19–29]	27% [24–32]	28% [23–47]	24% [19–29]	27% [24–32]	28% [23–47]	24% [19–29]	27% [24–32]	28% [23–47]
Population currently smoking (%)	25% [22–32]	24% [21–28]	31% [20–32]	27% [24–30]	26% [23–30]	30% [27–33]	27% [24–30]	26% [23–30]	30% [27–33]
Population with obesity (%)	23% [21–25]	21% [18–25]	23% [21–24]	28% [26–29]	29% [28–29]	28% [26–28]	28% [26–29]	29% [28–29]	28% [26–28]
Population with hypercholesterolemia (%)	8% [5–8]	7% [6–8]	8% [4–8]	9% [4–10]	6% [5–10]	6% [4–9]	9% [4–10]	6% [5–10]	6% [4–9]
Health care access (number of hospital beds per 1,000 population)	11·4 [11·3–11·6]	11·6 [11·5–11·7]	11·4 [11·3–11·6]	11·6 [11·4–11·7]	11·7 [11·6–11·7]	11·6 [11·4–11·6]	12·1 [11·9–12·2]	12·1 [12·1–12·2]	12·0 [11·9–12·2]
Variability in daily outdoor temperature (°C, annual)	3·3 [3·1–3·4]	3·0 [3·0–3·2]	3·2 [3·1–3·4]	3·6 [3·3–3·8]	3·3 [3·1–3·4]	3·6 [3·5–3·8]	3·9 [3·6–4·1]	3·6 [3·3–3·7]	3·9 [3·8–4·1]

Variability in daily outdoor temperature (°C, heating season)	266 [225–350]	325 [277–383]	248 [224–286]	281 [234–339]	326 [269–373]	258 [216–312]	236 [211–298]	278 [252–310]	223 [194–290]

Notes: Eligible townships were considered as "exposed" to the CHP if over 50% of their villages were participating in the policy. *Unexposed* refers to the group of townships that were not exposed to the CHP throughout the study period. *Exposed in 2016-2017* refers to the group of townships that were first exposed to the CHP in 2016-2017. *Exposed in 2018-2019* refers to the group of townships that considered unexposed in 2016-2017 and first exposed to the CHP in 2018-2019. Heating season refers to the months of December, January, and February. Obesity was defined as a BMI ≥28kg/m^2. Hypercholesterolemia was defined as total cholesterol ≥6·22 mmol/L.

Table S3: Average treatment effect on the treated (ATT) of the Clean Heating Policy (CHP) on acute myocardial infarction (AMI) incidence Beijing townships: Results from multiple sensitivity analyses. The ATTs are presented as the percent change in AMI events per 100,000 population with 95% confidence intervals in brackets

Age-sex category	Main analysis	Age standard-ization (35-49y, 50-64y, 65-79y, 80y+)	Oldest age groups (65-79y, and 80y+)	Two-year AMI rates estimated for Nov to Oct	One-year AMI rates estimated for Jan to Dec	Excluding two outlier townships	Excluding potentially ineligible townships	CHP-exposure defined as >70% of villages in the townships exposed to the policy. Unexposed is defined as <30%	Adjusting for hyper-cholesterolemia	Adjusting for variability in heating season temperature
All adults	-6·6<br>[-12·3, -0·8]	-7·0<br>[-12·2, -1·4]	..	-5·7<br>[-10·6, -0·6]	..	-6·6<br>[-12·3, -0·9]	-5·8<br>[-12·6, 1·5]	-8·1<br>[-17·9, 2·5]	-5·1<br>[-10·9, 0·8]	-5·0<br>[-12·0, 2·5]
Men	-3·3<br>[-9·5, 3·3]	..	..	-3·5<br>[-9·2, 2·6]	..	-3·4<br>[-9·7, 2·1]	-3·6<br>[-11·7, 5·3]	-6·9<br>[-18·0, 5·4]	-2·2<br>[-8·6, 4·6]	-0·5<br>[-7·8, 7·5]
Women	-11·7<br>[-19·0, -4·1]	..	..	-8·2<br>[-15·5, -0·6]	..	-11·4<br>[-17·2, -3·3]	-7·5<br>[-14·7, 0·0]	-5·9<br>[-17·1, 6·4]	-9·0<br>[-16·7, -0·7]	-11·4<br>[-20·3, -1·8]
65y+	-10·7<br>[-17·4, -3·6]	-10·3<br>[-17·0, -3·3]	..	-8·6<br>[-15·1, -1·6]	-7·3<br>[-18·6, 5·3]	-10·4<br>[-17·2, -3·3]	-9·5<br>[-17·0, -1·5]	-8·1<br>[-19·5, 4·8]	-9·2<br>[-16·6, -1·5]	-11·2<br>[-19·8, -2·0]
Men 65y+	-7·3<br>[-14·3, 0·1]	..	..	-6·2<br>[-14·0, 2·2]	-6·2<br>[-17·2, 6·2]	-7·1<br>[-14·1, 0·4]	-7·8<br>[-15·9, 0·8]	-10·8<br>[-22·8, 2·7]	-7·1<br>[-15·3, 1·6]	-7·3<br>[-15·9, 1·9]
Women 65y+	-13·3<br>[-22·4, -3·4]	..	..	-10·2<br>[-19·0, -0·6]	-8·5<br>[-21·0, 5·6]	-13·1<br>[-22·2, -3·1]	-9·9<br>[-18·9, 0·0]	-3.2<br>[-18·2, 14·4]	-10·5<br>[-20·2, 0·1]	-14·4<br>[-25·0, -2·5]
65-79y	..	..	-8·9<br>[-16·1, -1·2]	..	..	..	..	..	..	..
80y+	..	..	-10·9<br>[-21·5, 1·0]	..	..	..	..	..	..	..

Notes: Townships were considered "exposed" to the CHP when over 50% of villages in the township were participating in the policy and otherwise as "unexposed". Effect estimates are presented as the percent change in AMI incidence, calculated as (exp[coef]−1)*100 to account for log-transformed AMI rates. Covariate-adjusted analyses included the township proportion of population working in agriculture, the proportion of population having completed secondary school education or higher, the proportion of population unemployed, the proportion population reporting to currently smoking, the proportion population with obesity, exposure to a retired coal-fired power plant, variability in daily annual outdoor temperature, and health care access.

References

1. Wen H-x, Nie P-y, Liu M, et al. Multi-health effects of clean residential heating: evidences from rural China's coal-to-gas/electricity project. *Energy for Sustainable Development.* 2023; **73**: 66-75
2. Barrington-Leigh C, Baumgartner J, Carter E, Robinson BE, Tao S, Zhang Y. An evaluation of air quality, home heating and well-being under Beijing's programme to eliminate household coal use. *Nature Energy.* 2019; **4**(5): 416-23
3. Liu C, Zhu B, Ni J, Wei C. Residential coal-switch policy in China: development, achievement, and challenge. *Energy Policy.* 2021; **151**: 112165
4. Xu X, Dong J, Zhong X, Xie D. Climate impact of coal-to-clean-energy shift policies in rural Northern China. *Journal of Cleaner Production.* 2024; **434**: 139870
5. Hu Z. De-coalizing rural China: a critical examination of the coal to clean heating project from a policy process perspective. *Frontiers in Energy Research.* 2021; **9**: 707492
6. National Bureau of Statistics of the People's Republic of China. http://www.stats.gov.cn/tjsj/tjbz/tjyqhdmhcxhfdm/2019/11.html. (Date last accessed December 17, 2020)
7. Baidu Maps. http://map.baidu.com. (Date last accessed February 2, 2023)
8. Baidu Baike. https://baike.baidu.com. (Date last accessed February 2, 2023)
9. Cui C, Zuo X, Wang Y, Song H, Shi J, Meng K. A comparative study of patients' satisfaction with different levels of hospitals in Beijing: why do patients prefer high-level hospitals? *BMC Health Services Research.* 2020; **20**: 1-11
10. Besag J, York J, Mollié A. Bayesian image restoration, with two applications in spatial statistics. *Annals of the institute of statistical mathematics* 1991; **43**: 1-20.
11. Lee D. A comparison of conditional autoregressive models used in Bayesian disease mapping. *Spatial and Spatio-temporal Epidemiology.* 2011; **2**(2): 79-89
12. National Bureau of Statistics of the People's Republic of China. The sixth national population census of China. *China Statistics Press*. 2011
13. Chen X, Yang T, Wang Z, Hao Y, He L, Sun H. Investigating the impacts of coal-fired power plants on ambient $PM_{2.5}$ by a combination of a chemical transport model and receptor model. *Science of the Total Environment.* 2020; **727**: 138407
14. Global Energy Monitor. Global coal plant tracker. https://globalenergymonitor.org/projects/global-coal-plant-tracker. 2024; (Date late accessed June 28, 2024)
15. Chang J, Deng Q, Hu P, et al. Geographic variation in mortality of acute myocardial infarction and association with health care accessibility in Beijing, 2007 to 2018. *Journal of the American Heart Association.* 2023; e029769
16. Jin CD, Kim MH, Lee KM, Yun S-C. Effect of temperature variation on the incidence of acute myocardial infarction. *Journal of Korean Medical Science.* 2024; **39**(10)
17. Zhang N, Cao P, Zhao L, Wang L, Shao W, Li R. Effect of temperature fluctuations in cold seasons on acute myocardial infarction hospitalisations in northeast China: a retrospective observational cohort study. *BMJ Open*, 2023; **13**: e073528
18. Hersbach H, Bell B, Berrisford P, et al. ERA5 hourly data on single levels from 1979 to present. *Copernicus Climate Change Service (c3s) Climate Data Store (cds).* 2023; **10**(10.24381)
19. Callaway B, Sant'Anna PH. Difference-in-differences with multiple time periods. *Journal of Econometrics.* 2021; **225**(2): 200-30
20. Callaway B, Sant'Anna PH. did: Difference in Differences. 2021
21. Baker AC, Larcker DF, Wang CC. How much should we trust staggered difference-in-differences estimates? *Journal of Financial Economics.* 2022; **144**(2): 370-95
